# KCNQ Channels Show Conserved Ethanol Block and Function in Ethanol Behaviour

**DOI:** 10.1371/journal.pone.0050279

**Published:** 2012-11-29

**Authors:** Sonia Cavaliere, John M. Gillespie, James J. L. Hodge

**Affiliations:** School of Physiology and Pharmacology, University of Bristol, Bristol, Avon, United Kingdom; University of Waterloo, Canada

## Abstract

In humans, KCNQ2/3 channels form an M-current that regulates neuronal excitability, with mutations in these channels causing benign neonatal familial convulsions. The M-current is important in mechanisms of neural plasticity underlying associative memory and in the response to ethanol, with KCNQ controlling the release of dopamine after ethanol exposure. We show that dKCNQ is broadly expressed in the nervous system, with targeted reduction in neuronal KCNQ increasing neural excitability and KCNQ overexpression decreasing excitability and calcium signalling, consistent with KCNQ regulating the resting membrane potential and neural release as in mammalian neurons. We show that the single KCNQ channel in *Drosophila* (dKCNQ) has similar electrophysiological properties to neuronal KCNQ2/3, including conserved acute sensitivity to ethanol block, with the fly channel (IC_50_ = 19.8 mM) being more sensitive than its mammalian ortholog (IC_50_ = 42.1 mM). This suggests that the role of KCNQ in alcohol behaviour can be determined for the first time by using *Drosophila*. We present evidence that loss of KCNQ function in *Drosophila* increased sensitivity and tolerance to the sedative effects of ethanol. Acute activation of dopaminergic neurons by heat-activated TRP channel or *KCNQ-RNAi* expression produced ethanol hypersensitivity, suggesting that both act via a common mechanism involving membrane depolarisation and increased dopamine signalling leading to ethanol sedation.

## Introduction

Voltage-gated potassium (Kv) channels form a diverse gene family that, in humans, is subdivided into 12 subfamilies of 40 members [1]. Furthermore, functional Kv channels are tetramers, with multiple members of each individual subfamily able to form homo- or hetero-multimers with different properties. Such a diversity of channel types in mammals has made studying these channels in native tissue challenging; determination of the functional consequence of removal of a given channel at the whole organism level is often difficult due to genetic redundancy and compensation. Developing viable genetic models to study individual channel function is becoming increasingly important clinically, with mutations in over 60 channel genes resulting in channelopathies [2]. A potentially powerful approach is to use the genetics of *Drosophila*, which typically contains a single member of each Kv channel subfamily, with nulls being adult viable; this allows exploration of the functional consequence of complete lack of a subfamily of Kv channel [3].

KCNQ (Kv7) channels mediate a range of important physiological functions, form a hotspot of genetic diseases and are targets for new and existing drug treatments. In human cardiac muscle, *KCNQ1* mutations cause Long and Short QT [1], [2]. *KCNQ1* mutations also result in adult onset type II diabetes [4], [5]. In the nervous system, KCNQ2 and KCNQ3 heteromultimerise to form a channel that mediates the M-current and regulates membrane excitability in the sub-threshold range for action potential generation. Therefore, reducing neuronal KCNQ is usually sufficient to increase excitability of most neurons, with the M-current mediating changes in excitability that occur during synaptic plasticity and memory, alcohol response and nociception [1], [6], [7]. *KCNQ2/3* loss-of-function mutations result in a form of epilepsy. *KCNQ4* loss-of-function mutations cause autosomal dominant deafness. M-current inhibitors increase excitability and have shown some promise in enhancing memory in models of dementia. Conversely, M-current openers are of great interest as anticonvulsants, analgesics and treatments of psychiatric diseases [1], [8].

*Drosophila* has a single KCNQ channel (dKCNQ) that is most highly expressed in the nervous system [9], [10] but, like mammalian KCNQ1 [1], [2], is also expressed in the heart. dKCNQ encodes a slowly activating and deactivating Kv current that can be suppressed by muscarinic receptor agonists and hence is an M-current [10], [11]. dKCNQ has been shown to have an important role in age-dependent cardiac function, with *dKCNQ* loss-of-function mutations resulting in heart arrhythmia in young flies. This phenotype is also observed in aged wildtype flies, which correlates with an age-dependent decline in *dKCNQ* expression [9]. However, no neuronal characterisation of dKCNQ has been presented to date. *Drosophila* is a powerful model of the molecular and neuronal mechanisms of alcohol and other addictive drug-related behaviours, with many of these genes and mechanisms identified in *Drosophila* and then validated in mammals [12], [13]. We show that *Drosophila* and rat KCNQ2/3 channels are acutely sensitive to block by low concentrations of ethanol. We characterise for the first time the *in vivo* consequence of *dKCNQ* mutations on neural activity and behaviour, showing a role for the channel in regulation of ethanol sensitivity and tolerance.

## Materials and Methods

### DNA reagents

*Drosophila KCNQ RE26469* cDNA (Flybase FBgn0033494, vector: *pIRES2-EGFP*), rat *KCNQ2* cDNA (GenBank AAC36722; *pcDNA3.1*) and rat *KCNQ3* cDNA (AC79846; *pcDNA3.1*) [11]. Genomic database searches were performed with *Drosophila* RE26469 full-length KCNQ cDNA using the WU-BLAST server at EMBL-EBI.

### Cell culture

cDNAs were expressed in Human Embryonic Kidney (HEK293) cells using previously published protocols [11].

### Electrophysiology and pharmacology

Whole-cell voltage-clamp recordings were made from HEK293 cells using an Axopatch 200A amplifier (Axon Instruments, Molecular Devices, Sunnyvale, California, US) as previously described [11]. To determine the effect of ethanol at a depolarised voltage, currents were elicited by a single pulse protocol in which membrane potential was held at −80 mV for 100 ms, stepped to +30 mV for 1 s and stepped down to −120 mV for 250 ms. To generate I–V and G–V relations, the following multi-step protocol was used: membrane potential was held at −80 mV for 100 ms, stepped in increments of 10 mV from −80 mV to +30 mV then, after 1 s, stepped down to −120 mV for 250 ms. Currents were measured at the end of the sweep at maximal current for each step. I–V relations were plotted as normalised current in pA/pF (± standard error of the mean (SEM)) against voltage. The V_0.5_ value was the voltage required for half the maximal activation current. Mean V_0.5_ values are shown ± SEM. Data were analysed using Graphpad Prism.

### *Drosophila* stocks

The *KCNQ* deletion mutant contains an imprecise excision of the *EP2074* element (*KCNQ^186^*) that removes all the 5′ and transmembrane regions of the channel and therefore is likely a null mutation [9]. The *KCNQ* control was a precise excision of the element (*KCNQ^97^*), leaving the gene completely intact [9]. *uas-KCNQ* flies allowed *Gal4* promoter-driven overexpression of *KCNQ* [9], while *uas-KCNQ-RNAi* (Bloomington stock 27252) allowed *Gal4*-targeted knockdown of the channel. Wild-type flies were *Canton S w-* (*CSw-*) from a stock previously maintained in the Griffith lab. All *KCNQ* mutant, *Gal4* and *uas* lines were out-crossed with this *CSw-* line prior to behavioural analysis. All genotypes and all other crosses were raised on corn-meal malt-molasses agar medium at 22±2°C and 60±10% humidity under a 12∶12 h light-dark cycle.

### Immunohistochemistry

Adult fly brains or third-instar larvae were dissected in HL3.1 (70 mM NaCl, 5 mM KCl, 10 mM NaHCO_3_, 115 mM sucrose, 4 mM MgCl_2_ 5 mM trehalose, 1.5 mM CaCl_2_, and 5 mM HEPES, pH 7.3), and isolated brains from either stage were fixed in 4% paraformaldehyde in HL3.1 for 30 min before being washed in HL3.1 [14]. The preps were permeabilised in HL3.1 with 0.1% triton X (HL3.1-Tx) for 1 h, and then blocked for 1 h in HL3.1-Tx with 0.1% BSA and 2% normal donkey serum (HL3.1-Tx-BSA-NDS). To visualise the *vGlut(OK371)-Gal4* pattern, a (1∶1000) rabbit anti-*Drosophila* Glut antibody was used [15] overnight at 4°C in HL3.1-Tx-BSA-NDS. After washing three times in HL3.1-Tx for 20 min, the brains were incubated with anti-rabbit Alexa-648 conjugated secondary antibody (1∶400 in HL3.1-Tx-BSA-NDS) for 2 h at room temperature. Finally, the brains were washed three times in HL3.1-Tx before being mounted in Vectashield (Vector Laboratories). Samples were stored at 4°C in the dark until examination with a Leica TCS SP5 confocal microscope. The endogenous *KCNQ* expression pattern was determined by visualising membrane-targeted GFP expressed using *KCNQ-Gal4* reporter lines (*KCNQ^NP3423^-Gal4, uas-mCD8-GFP*).

### Calcium imaging

Third-instar larvae were dissected in haemolymph (HL3.1) solution containing 1.5 mM Ca^2+^ and were subsequently imaged using previously described methodology [16], [17]. Spontaneous Ca^2+^ signalling during peristaltic crawling was imaged at ten frames/sec in the A5 ventral ganglion motor neurons of larvae expressing GCaMP3, using the following setup: 10× water-immersion lens of a Zeiss Examiner Z1 microscope with LED, filters and AV4.8 software, with an Axiocam MRm camera system optimised for GCaMP3 imaging. A region of interest was drawn around the cell bodies of MN1-Ib, MN14-Ib, MN6/7-Ib, MN30-Ib and MNISN-Is in order to calculate the per cent increase in intensity above baseline (which was defined as the average of the first ten frames) for each time point. Images were processed using Volocity (PerkinElmer) and Image J (NIH). Data were statistically analysed and presented with Graphpad Prism software.

### Ethanol behavioural assays

All experiments were performed at 25°C and 70% humidity under white light. Twenty synchronised 3–5 day old male and female flies were used. One millilitre of 40% ethanol solution was added to an absorbent pad at the bottom of a sealed bottle. Active flies initially remained on the walls or up beneath the bottle lid. During the test period, a number of flies became anesthetised and were immobilised at the bottom of the bottle. These flies were counted at 5-min intervals over the 90-min ethanol exposure to measure sensitivity [18]. To test the effect of acute activation of dopamine neurons on ethanol sensitivity, flies expressing the heat-activated TRPA1 channel in these neurons were tested at either 23°C or 30°C, with only the higher temperature sufficient to cause TRPA1 activation and depolarisation [19]. To measure tolerance, flies were given a first ethanol exposure as above and then allowed to recover for 2 h on food without ethanol. The flies' response to the same ethanol exposure a second time was then measured [18]. Flies recovered fully after the ethanol exposures used in these experiments. Data were analysed using Graphpad Prism.

### Ethanol content and preference assay

Ethanol content of flies of different genotypes was determined by exposing twenty active flies to ethanol vapour in a sealed bottle that an absorbent pad soaked in 1 ml of 40% ethanol. Flies were frozen in liquid nitrogen and stored at −80°C. Flies were homogenised with 500 µl of Tris-HCl (pH 7.5) and spun at 4°C for 20 min at maximum speed. The clear supernatant was recovered and used as the test sample. The alcohol reagent kit (273-30, Genzyme) was used to measure total ethanol content using the ADH-NADH reagent test [18]. Ethanol avoidance was quantified with a T-maze test in which a solution of 40% ethanol was placed in the odour cup of one arm of the T-maze during a 2-min trial. The performance index was calculated by counting the number of flies avoiding 40% ethanol divided by total number flies. All statistical analyses for behavioural data were performed and plotted with Graphpad Prism software.

### Semi-quantitative RT-PCR

Total RNA was isolated from adult fly heads using a Trizol solution (Invitrogen). An equal number of age-matched control flies were frozen in liquid nitrogen and decapitated by vortexing. The detached heads were collected and homogenised. Trizol was added directly to the homogenised heads and RNA was extracted by ethanol precipitation. RNA was DNase-treated (Ambion, Inc.) and reverse-transcribed using a first-strand complementary DNA (cDNA) synthesis kit (RevertAid, Fermentas). The first strand cDNA was obtained from a total RNA template in a single reaction, adding a reverse transcriptase enzyme and oligo (dT) primers and incubating for 1 h at 42°C [9]. PCR reactions were performed as follows: 4 min at 95°C; then 30 s at 95°C, 60 s at 60°C, and 2 minutes at 72°C for 25 cycles; followed by a final 7 min at 72°C. All primers were optimally designed and synthesised by Invitrogen.

KCNQ forward: 5′-AGGAAAGCCGCTGAACTACA-3′, position 210–230,

KCNQ reverse: 5′-CGAGGTGCCCATTCCTAATA-3′, position 604–584.

The PCR amplification product was analysed by electrophoresis in 1% agarose gels and visualised by ethidium bromide staining and transillumination under UV light.

## Results

### The level of KCNQ regulates neural excitability *in vivo*

**Figure 1:**
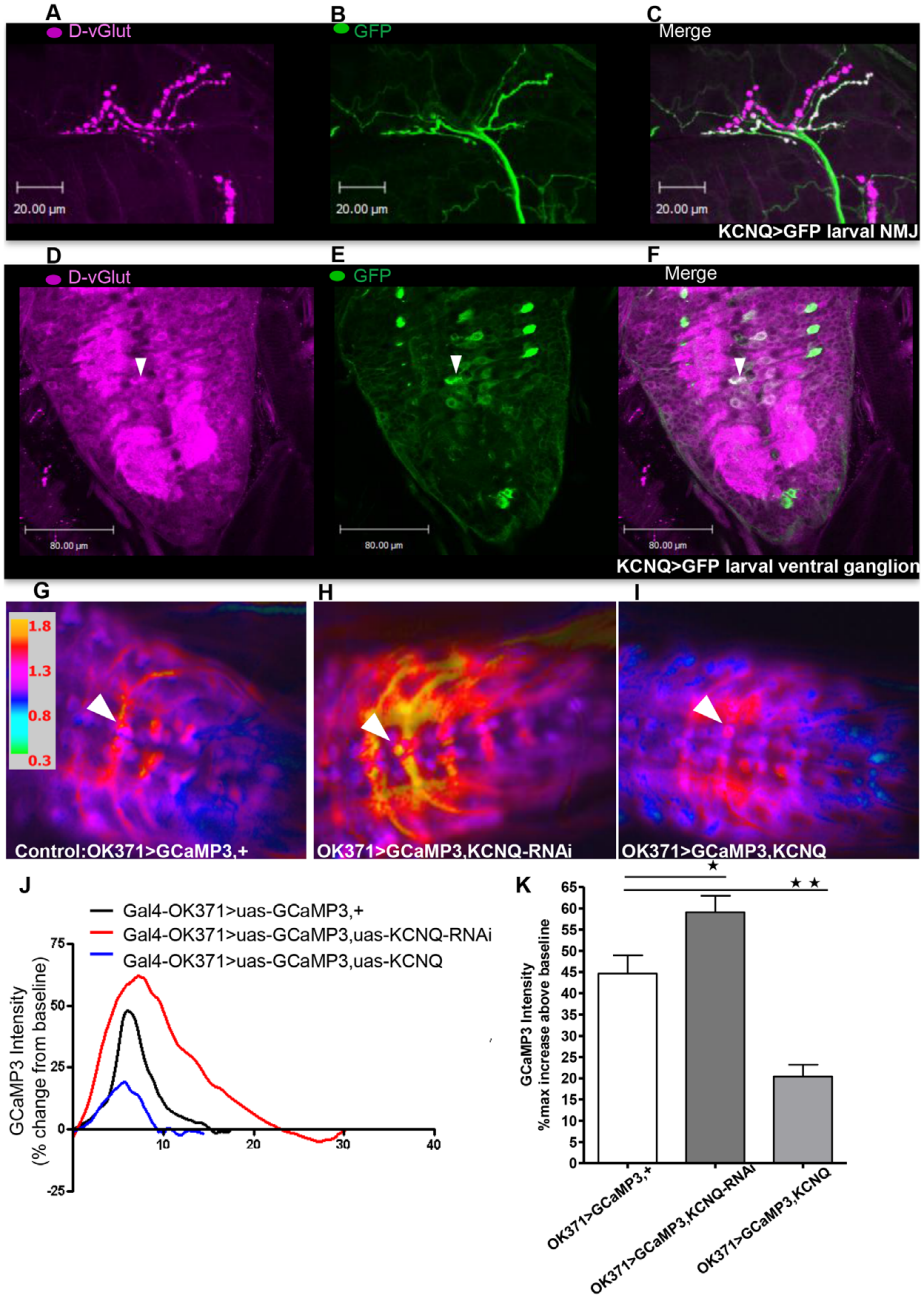
The level of KCNQ sets neural excitability. **A–F.** *KCNQ* is expressed in the motor neurons that were measured in the GCaMP3 experiments. Larvae containing a *Gal4* enhancer trap (*KCNQ^NP3423^*) in the *KCNQ* gene locus show high expression of *KCNQ* (labelled by membrane bound GFP in green, B and E) in the nervous system and particularly in glutamatergic motor neurons as visualised by the *Drosophila* vesicular glutamate transporter (vGlut) antibody stain (in magenta, A and D). Co-expression of vGlut and KCNQ is shown in white in the motor neuron cell bodies and neuropil in the ventral ganglion (F) and respective neuromuscular junctions (NMJs) (C) and particularly in the motor neuron cell body (white arrow head) measured in the GCaMP3 experiments. These motor neurons were labelled by the *vGlut(OK371)-Gal4* promoter [15] used in the GCaMP experiments. As this *Gal4* element is inserted in the *vGlut* locus, a vGlut antibody stain (in magenta, A and D) can be used to show co-expression of a gene (such as *dKCNQ*) in the *Gal4-OK371* expression pattern [15]. G–K. Spontaneous Ca^2+^ signalling was imaged using *Gal4-OK371*, *uas-GCaMP3*. Representative ratiometric images of larvae of control (*Gal4-OK371*, *uas-GCaMP*, *+*) (**G**.); *Gal4-OK371*, *uas-GCaMP*, *uas-KCNQ-RNAi* (**H**.); and *Gal4-OK371*, *uas-GCaMP*, *uas-KCNQ* (**I**.) showing their maximum increase in fluorescent intensity above baseline, with the specific motor neuron soma used for measurements shown by the white arrowhead. **J**. Sample traces showing the time course of the bidirectional change in GCaMP3 signal with changes in neuronal KCNQ level. **K**. Histogram showing that *KCNQ-RNAi* (dark grey bar in this and subsequent figures) expression in motor neurons increases (p<0.05) the amplitude of Ca^2+^ influx, whereas *KCNQ* overexpression (light grey bar in this and later figures) decreases (p<0.01) the amplitude when each is compared with control (white bar in this and other figures, n>4). Data were analysed by 1-way ANOVA with a Bonferroni post-hoc test (n≥8).

To determine how KCNQ may affect neural excitability, we first identified the neurons in which KCNQ is expressed. Consistent with previous reports [9], [10], KCNQ was found to be broadly expressed in the embryonic nervous system (Figure S1). To investigate expression later in development, a *Gal4* enhancer trap within the *KCNQ* gene was used to express GFP. These experiments revealed that KCNQ continues to be broadly expressed in the nervous system, including in glutamatergic motor neurons (Figure 1B and E) that also strongly express the *Drosophila* vesicular glutamate transporter (D-vGlut, [15]) (Figure 1A and D). We therefore employed the *vGlut(OK371)-Gal4* line that has particularly strong motor neuron expression in larvae. These neurons also express *KCNQ* (Figure 1C and F). In the adult brain, *KCNQ* appears to be broadly expressed, including in neurons in and around the mushroom body (unpublished data). To explore how the level of KCNQ may be affecting neural excitability, we performed *in vivo* Ca^2+^ imaging in *Drosophila*. We used *vGlut(OK371)-Gal4* to express a Ca^2+^ reporter, GCaMP3 [16], to examine neural activity in the KCNQ-expressing neurons. During larval peristalsis, there is a wave of Ca^2+^ signalling that travels through the ventral ganglion. We measured the maximum change in fluorescence in the motor neuron soma when this occurs. Expression of *KCNQ-RNAi* caused a significant increase in neuronal excitability (Figure 1H, J and K) compared with control (Figure 1G). Overexpression of the KCNQ channel reduced Ca^2+^-induced fluorescence and hence neural excitability (Figure 1I, J, K). These bidirectional changes in KCNQ expression and neural excitability might be expected to interfere with neural release, as has been demonstrated in other systems [1], [6]. Expression of *KCNQ* or *KCNQ-RNAi* in dopa-decarboxylase (Ddc) neurons resulted in a wing-expansion phenotype (Figure S2); such a phenotype has previously been reported to occur as a result of large changes in neuronal hyper- or de-polarisation, disrupting the neuropeptide release required for wing expansion [20]–[22].

### dKCNQ current is similar to the neuronal M-current encoded by KCNQ2/3, with both showing ethanol block

**Figure 2:**
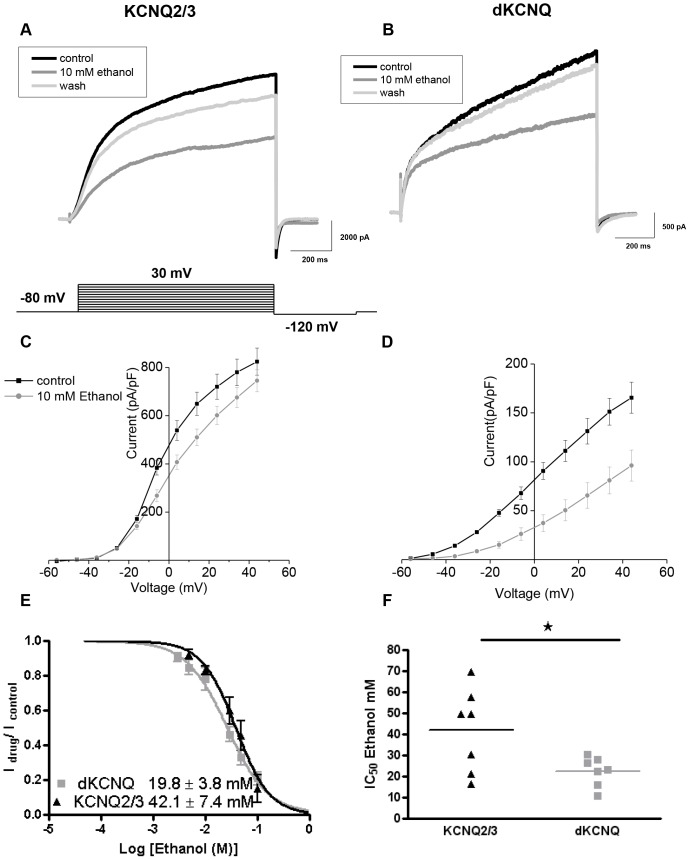
KCNQ2/KCNQ3 and dKCNQ are acutely sensitive to ethanol inhibition. Representative traces recorded from HEK cells (**A**) showing that KCNQ2/3 current (black) was blocked by 10 mM ethanol (grey), as seen by the downward shift of the I–V relation (**C**) after ethanol application. The ethanol block was reversible (light grey traces in A). Representative traces (**B**) and I–V relation (**D**) showing acute reversible block of dKCNQ by ethanol. **E.** Plot showing the effect of increasing concentrations of ethanol on dKCNQ (grey) and KCNQ2/3 (black) currents. The maximum peak current amplitude evoked at 30 mV at the end of the 1350 ms pulse with or without ethanol was compared. **F**. Comparison of IC_50_s showing that dKCNQ is more sensitive (p<0.05) to ethanol inhibition than is KCNQ2/3 (n = 7). Data were analysed by Student's unpaired *t*-test. For all figures: error bars are standard error of the mean and no asterisk means not significant; *p<0.05, **p<0.01 and ***p<0.001.

Ethanol has recently been demonstrated to inhibit the M-current in dopaminergic neurons of the ventral tegmental area (VTA, [6]); however, neither the molecular identity of the channel subunits nor the effect of ethanol on KCNQ currents was studied. Therefore, we expressed mammalian KCNQ2/3 in Human Embryonic Kidney (HEK) cells and found that application of low concentrations (10 mM) of ethanol caused acute block of the KCNQ2/3 current at +30 mV (Figure 2A and C). Ten-millimolar ethanol has little effect on the KCNQ2/3 activation curve (Figure S3A), although high concentrations of ethanol could conceivably change the channel's gating. The IC_50_ for the ethanol block of KCNQ2/3 was 42.1±7.4 mM (Figure 2E–F). Similarly to KCNQ2/3, the dKCNQ carries a slowly activating and non-inactivating Kv current that opens at sub-threshold potentials for action potential generation (Figure 2). Under the same conditions, dKCNQ current was sensitive to ethanol block (Figure 2B and D), with little effect on the activation curve (Figure S3B) and an IC_50_ of 19.8±3.8 mM, which is significantly lower than that of mammalian KCNQ2/3 (Figure 2E–F).

### Neuronal KCNQ regulates ethanol sensitivity in *Drosophila*

**Figure 3:**
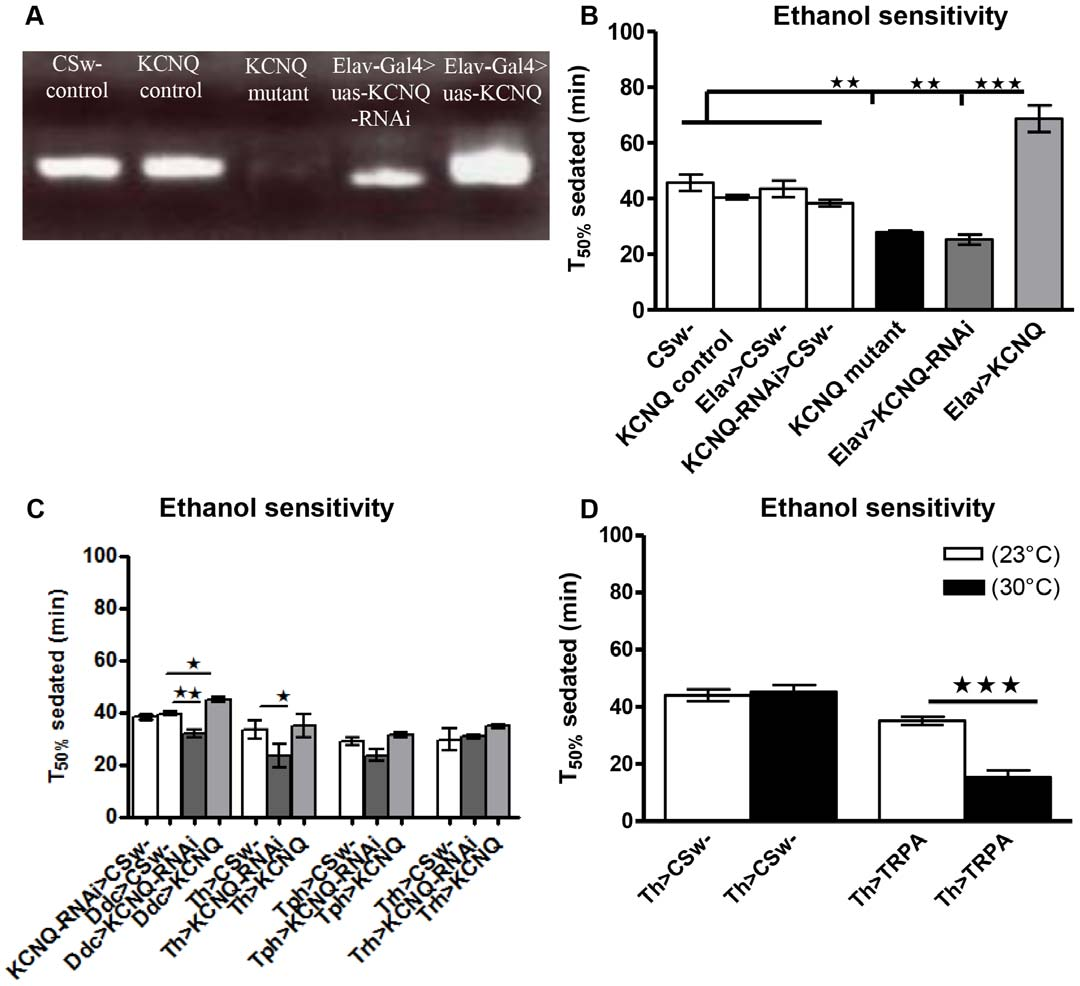
KCNQ in dopaminergic neurons regulates ethanol sensitivity behaviour. **A**. Ethidium-bromide-stained agarose gel containing products of semi-quantitative PCR, showing removal of *KCNQ* in the *KCNQ* mutant, reduced *KCNQ* from flies expressing *KCNQ-RNAi* in all neurons (*Elav-Gal4*) and increased *KCNQ* from flies overexpressing *KCNQ* in all neurons compared to controls (*CSw-* as wild-type and *KCNQ* control as the precise excision of the *P*-element). **B**. *KCNQ* loss-of-function *P*-element mutant flies (black bar in this and other figures, unless otherwise stated) or flies with pan-neural reduction in *KCNQ* expression (*Elav-Gal4, uas-KCNQ-RNAi*) exhibit a similar increase (p<0.01) in sensitivity to 40% ethanol compared with controls: *CSw-* wild-type (*+*), the *KCNQ* precise excision (*KCNQ* control), *Elav-Gal4*, *+* and *uas-KCNQ-RNAi*, *+*. Conversely pan-neural *KCNQ* overexpression (*Elav-Gal4, uas-KCNQ*) caused ethanol resistance (p<0.001). These changes in sensitivity were quantified as time to sedate 50% (T_50%_), the time taken for half of the flies of a given genotype to become sedated. **C**. Reduction of *KCNQ* in dopamine and serotonin neurons (*Ddc-Gal4, uas-KCNQ-RNAi*) increases sensitivity (p<0.01), while overexpression (*Ddc-Gal4, uas-KCNQ*) decreases sensitivity (p<0.05) with respect to control *Ddc-Gal4*, *+*. Reducing *KCNQ* in dopamine neurons (*Th-Gal4, uas-KCNQ-RNAi*) was sufficient to cause the increase (p<0.05) in ethanol sensitivity compared with control (*Th-Gal4, +*) while manipulating *KCNQ* levels in serotonin (*Tph-* or *Trh-Gal4*) neurons alone had little effect (p>0.05). Data in B–C were analysed by 1-way ANOVA with a Bonferroni post-hoc test (n>4, 20 flies per n). **D**. The ethanol sensitivity of *Th-Gal4, +* and *Th-Gal4*, *uas-TRPA1* flies was measured at 23 or 30°C (n>6, 20 flies per n). Two-way ANOVA indicates significant differences in sensitivity due to interaction between temperature and genotype (p<0.0001). Post-hoc analysis showed that acute depolarisation by heat-activated (30°C) TRPA1 causes hypersensitivity (p<0.0001) when expressed in Th neurons, whereas the sensitivity of *Th-Gal4*, *uas-TRPA1* flies at 23°C was similar to control animals at 23 or 30°C.

As ethanol reduced the KCNQ current, we proceeded to determine if *KCNQ* loss-of-function would further increase the fly's sensitivity to ethanol, the rationale being that they had been made more susceptible to ethanol due to their lack of functioning KCNQ channels. We employed an assay that involved exposing flies to 40% ethanol vapour and scoring sedation, whereas active flies initially remain on the walls and beneath the lid of the bottle. During the ethanol exposure, flies become sedated and are immobilised at the bottom of the bottle. These flies were counted at 5-min intervals over the ethanol exposure in order to measure sensitivity to the sedative effects of ethanol ([18], Figure 3). Wild-type flies (Figure 3B, *KCNQ* control) show an increase in sedation over 90 min. A *KCNQ* loss-of-function *P*-element mutant with greatly reduced *KCNQ* (Figure 3A) showed ethanol hypersensitivity (Figure 3B) compared with flies with a precise excision that leaves the gene intact and is the *KCNQ* control [9]. Pan-neural reduction in *KCNQ* (Figure 3A) resulted in a similar ethanol hypersensitivity to the *KCNQ* mutant while *KCNQ* overexpression in all neurons (Figure 3A) resulted in a decrease in sensitivity (Figure 3B).

Prior work has shown that aminergic (dopamine and serotonin) neurons mediate the neural response to addictive drugs such as ethanol [6], [23]–[25]. In *Drosophila*, *Ddc-Gal4* expresses in both serotonin and dopamine neurons; this promoter has been used to change the expression of a number of genes that alter the function of these neurons and result in modulation of the ethanol behaviour of the fly [26]–[28]. Therefore, to investigate the neuronal subtypes that underlie the *KCNQ* behavioural phenotype, we targeted the reduction of *KCNQ* to Ddc neurons. This caused ethanol hypersensitivity (Figure 3C); conversely, *KCNQ* overexpression in Ddc neurons was sufficient to cause ethanol resistance. To further dissect the neural circuitry mediating KCNQ's role in ethanol sensitivity, we expressed *KCNQ-RNAi* specifically in dopamine neurons, using *Tyrosine Hydroxylase (Th)-Gal4* [28]. This was sufficient to cause ethanol hypersensitivity. In contrast, changing the level of KCNQ in serotonin neurons using tryptophan hydroxylase promoter lines (*Trh*- and *Tph-Gal4*, [26]) had little effect on ethanol behaviour.

We expressed the heat-activated *TRPA1*, a channel known to cause a large depolarisation of *Drosophila* neuronal membrane potential that triggers firing action potentials without substantial inactivation of Na^+^ channels [19], in the same Th (dopamine) neurons (Figure 3D). This was done both to investigate the importance of Th neuronal subtypes in mediating this alcohol behaviour and to understand the mechanism of action of *KCNQ-RNAi* in neurons. Heat activation of *Th-Gal4*, *uas-TRPA1* showed an ethanol hypersensitivity phenotype but did not cause any other non-specific locomotor defect (Figure S4A–B). This phenotype was also seen when *KCNQ-RNAi* was expressed in the same neurons, suggesting that both act via a common mechanism involving membrane depolarisation.

### KCNQ signalling regulates development of ethanol tolerance

**Figure 4:**
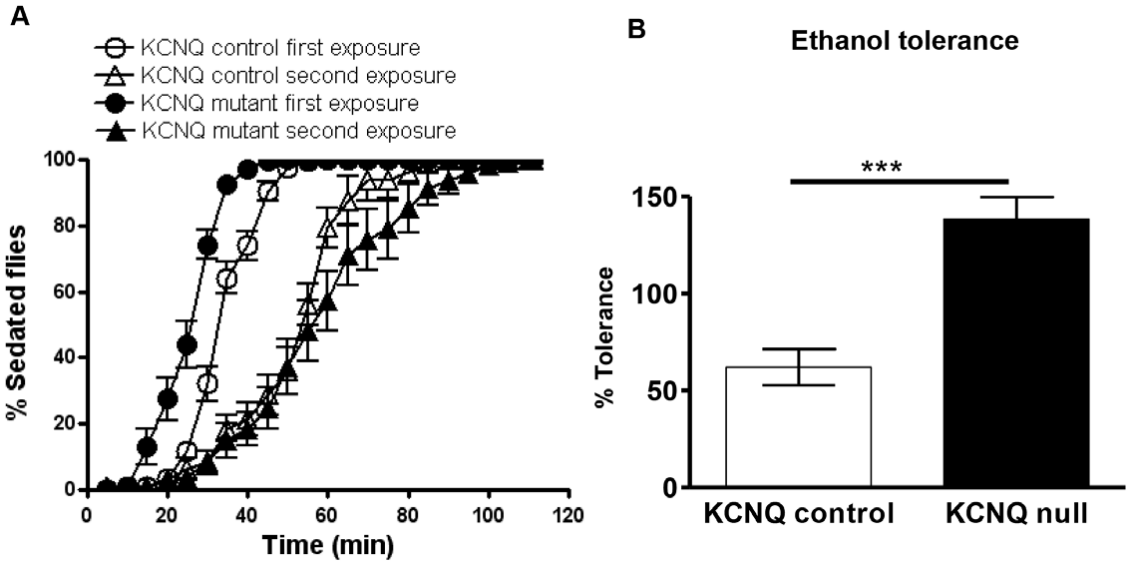
KCNQ channel levels regulate the development of ethanol tolerance. **A**. Flies were given two identical 90-min exposures to 40% ethanol vapour separated by a 2 h recovery; first (black circles) and second (black triangles) exposures of KCNQ mutants are separated by a greater rightward shift of the ethanol behavioural response curve (greater tolerance) than the shift between the controls' first (white circles) and second exposures (white triangles). **B**. Histogram showing these changes in tolerance quantified using the following equation: T_50%_ second exposure – T_50%_ first exposure/T_50%_ first exposure×100. KCNQ mutants (p<0.01) developed more tolerance than control (n = 10, 20 flies per n) as analysed by unpaired t-test.

Animal models such as *Drosophila* can be used to study specific aspects of human addiction, such as tolerance [12], [13]. KCNQ loss of function resulted in an ethanol tolerance phenotype (Figure 4); whereas on first ethanol exposure the mutant is more sensitive than control, on second exposure the mutant becomes less sensitive (Figure 3B and 4A). This resulted in a larger shift in the ethanol behavioural response curve (Figure 4A), a measure of tolerance which was greater in the KCNQ mutant than in the control (Figure 4B). To ascertain whether these changes in ethanol behaviour resulted from alterations in KCNQ neural signalling (as opposed to purely metabolic changes), we measured the ethanol content of the flies (Figure S4C). No difference in ethanol metabolism was found between genotypes, with circulating levels of ethanol ∼25 mM at the end of the exposure that were reduced to 10 mM after 50 min. Furthermore, all genotypes chose equally to avoid 40% ethanol vapour in the T-maze (Figure S4D).

### *Drosophila* display age-dependent ethanol hypersensitivity that is mimicked by KCNQ mutants

**Figure 5:**
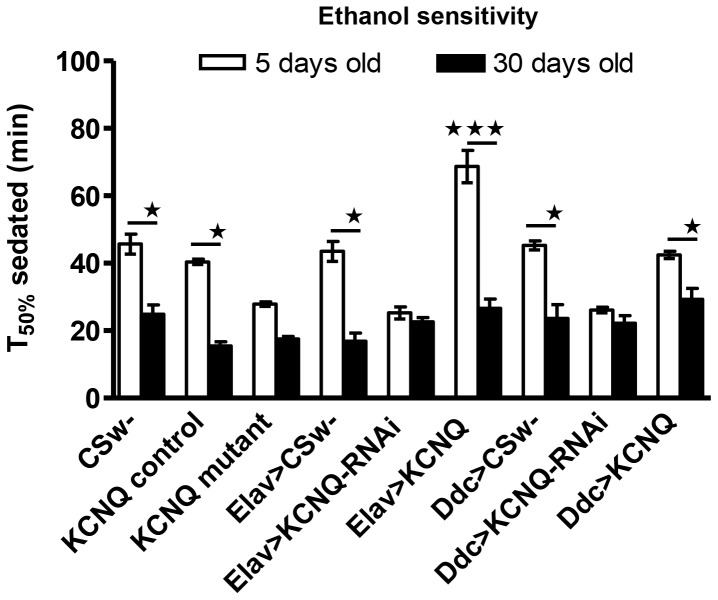
Ethanol sensitivity is similar between aged flies and *KCNQ* loss-of-function mutants. The sensitivity of flies to the sedative effect of 40% ethanol was compared between young (5 days) and aged (30 days, black bars) flies of the same genotype (n≥4, ∼100 flies per n). Two-way ANOVA indicates significant differences in memory due to interaction between age and genotype (p<0.0001). Post-hoc analysis showed that *CSw-*, *KCNQ* control, *Elav-Gal4/+*, *Ddc-Gal4/+*, *Ddc-Gal4/uas-KCNQ* (p<0.05) and *Elav-Gal4/uas-KCNQ* (p<0.001) flies were more sensitive to ethanol when old than young. The *KCNQ* mutant, *Elav-Gal4/uas-KCNQ-RNAi* and *Ddc-Gal4/uas-KCNQ-RNAi* showed no effect of age, being equally hypersensitive to ethanol when young or old (p>0.05).

KCNQ expression decreases in aged flies ([9], unpublished data), which would predict that old flies have would have increased ethanol sensitivity compared to young flies, in which KCNQ expression is higher. Therefore, we tested the effect of age on ethanol sensitivity and found that aged flies of a given genotype were more sensitive to ethanol than young flies (Figure 5), with the exception of the *KCNQ* mutants and flies expressing *KCNQ-RNAi* in Elav or Ddc neurons (which were already ethanol hypersensitive as young flies).

## Discussion

This study has characterised the electrophysiological properties of dKCNQ compared to mammalian neuronal KCNQ2/3 channels, showing that dKCNQ encodes a slowly activating and non-inactivating Kv current (Figure 2) similar to the M-current [10], [11]. We went on to determine the effect of ethanol on KCNQ channels. Ethanol has a number of molecular targets; for instance, ∼10 mM ethanol inhibits NMDA receptors and enhances GABA_A_ receptors [29], [30], while ∼100 mM ethanol opens GIRK and BK K^+^ channels but closes Shaw K^+^ channels [6], [30]–[32]. In this study, we show that the *Drosophila* KCNQ channel is more sensitive to ethanol than the mammalian channel, with an IC_50_ of 19.8 mM compared to 42.1 mM for KCNQ2/3 (Figure 2E–F). This may be because flies have evolved to prefer and to live off fermenting fruit; therefore, in order to adapt to their environmental niche, there may have been selection for genes regulating ethanol sensitivity and behavioural response to ethanol [33], [34]. The IC_50_ for ethanol on KCNQ2/3 is consistent with this heteromultimeric channel mediating the M-current that was also blocked by ethanol *in vivo* (20–120 mM; [6]). Furthermore, the KCNQ block seen here is likely to be physiologically relevant, as the blood alcohol content legally considered impairing is ∼20 mM [30]. Further investigation of the physiological consequence of this increased ethanol sensitivity of KCNQ in flies showed that ethanol caused sedation of wild-type flies with circulating ethanol levels of 25–30 mM (Figure S4C). This is comparable with intoxicating levels in humans and is sufficient to block the majority of KCNQ channels. *KCNQ* loss-of-function flies became sedated more quickly, demonstrating their increased ethanol sensitivity (Figure 3B).

Reducing *KCNQ* expression only in Ddc neurons caused hypersensitivity, while Ddc *KCNQ* overexpression resulted in resistance (Figure 3C). The KCNQ ethanol behaviour is consistent with our ethanol electrophysiology data, as exposure to comparable ethanol levels caused a reduction in KCNQ current. Loss-of-function alleles that further reduce the level of KCNQ in neurons made the fly even more sensitive to ethanol exposure. Conversely, neuronal *KCNQ* overexpression would be expected to cause a large hyperpolarisation. This might have the consequence that the depolarising effect of KCNQ ethanol blockade might have little effect, as the membrane potential would be sub-threshold for action potential firing or neurotransmitter release. Reduced *KCNQ* and the resultant depolarisation of Th neurons was sufficient to bring about this ethanol hypersensitivity, showing that dopaminergic neurons mediate KCNQ's effect on this behaviour (Figure 3C). We found that TRP activation and depolarisation of the Th neurons caused similar ethanol hypersensitivity (Figure 3D), confirming the importance of these neurons for this behaviour and suggesting that *KCNQ-RNAi* is likely to act via a common mechanism involving neuronal depolarisation. These results are consistent with dopamine being central for mediating the neural response to addictive drugs such as ethanol in human, mammalian and fly models [23]–[25], [27], [28], [35], [36]. They are also consistent with the M-current being specifically inhibited in dopamine neurons of the VTA, with ethanol likely to change the firing and release of the dopamine neurons [6]. Similarly, we found that changes in KCNQ levels in Ddc neurons resulted in a phenotype associated with impaired neural release (Figure S2F) and that reduced KCNQ increased neural excitability and overexpression decreased excitability *in vivo* (Figure 1).

Longer-term changes in response to repeated exposure to drugs such as ethanol are required to bring about addiction [13], [25]. These include functional changes in the nervous system, such as a decreased response to a given concentration of drug on repeated exposure, e.g., functional neuronal tolerance [12], [25]. *KCNQ* mutants also display increased ethanol tolerance compared to wildtype; therefore, functional KCNQ would decrease flies' sensitivity to and tolerance of ethanol. All of these ethanol phenotypes seem to be caused by functional neural adaptive changes involving KCNQ, as no changes in ethanol pharmacokinetics were detected between genotypes. Other major regulators of neuronal excitability, such as GABA_B_ receptors and BK channels, also affect fly ethanol sensitivity and tolerance [12], [37]. It is conceivable that these ethanol targets regulate the excitability of neural circuits in response to ethanol, thereby mediating part of the increase in ethanol sensitivity and tolerance seen in the KCNQ mutant. GABA_B_ receptors couple to GIRK channels, decreasing neural excitability [38], and GABA_B_ receptors usually promote ethanol sensitivity and tolerance in *Drosophila* [37], with ethanol also being able to directly bind and open GIRKs, decreasing excitability [39]. The role of BK in ethanol behaviour is more complex, with mammalian BK (Slo) channels being opened by ethanol and increasing tolerance by a number of mechanisms. In worm, *slo* mutations decrease ethanol sensitivity, while in flies, *slo* is transcriptionally induced by ethanol exposure, causing tolerance [12], [32].

Interestingly, work from humans and mammalian models has shown that initial sensitivity to ethanol can predict future ethanol consumption and alcoholism, with the same set of genes thought to underlie both [40], [41]. As KCNQ regulates both initial sensitivity to ethanol and subsequent development of tolerance, it is possible that KCNQ might be a candidate gene to predict susceptibility to alcoholism. This and recent work [42], [43] suggest that KCNQ openers may offer a potential new approach to treatment of alcohol- and other drug-misuse disorders. Behavioural changes underlying addiction involve associative memory, with a role for dopamine signalling in mediating reinforcement in both flies and mammals [24], [28], [44]. *Th-Gal4* expresses in dopamine neurons that innervate the mushroom body and mediate reinforcement in associative memory [28], [45]. Our data are consistent with a recent study showing that perturbing neurotransmission in Th neurons blocked a conditioned preference for ethanol, with pharmacological depletion of dopamine but not serotonin being sufficient to bring about this block [28]. *KCNQ* expression decreases in aged flies ([9], unpublished data) again correlating with the ethanol hypersensitivity phenotype (Figure 5).

Interestingly, optogenetic membrane depolarisation of dopamine neurons [46] recapitulated the initial increase in locomotor hyperactivity and subsequent long term sedation that is seen in *Drosophila* or mammals exposed to either long periods or dose-dependent increases in ethanol or cocaine [23], [35]. Given the conserved role of mammalian KCNQ2/3 in the neurophysiological response to ethanol, it is likely that genetic or pharmacological disruption of KCNQ will result in similar ethanol behaviour phenotypes in mammals. We have, therefore, validated the use of *Drosophila* to study KCNQ neuronal function and alcohol behaviour. The fly's compatibility with high-throughput analysis has the potential to allow both the identification of the underlying mechanism for this behaviour and screening for new therapies for alcohol related behavioural disorders and KCNQ diseases.

## Supporting Information

Figure S1 ***KCNQ*** **is widely expressed in the nervous system.** **A.** Stage 17 wild-type embryos hybridised with *KCNQ* antisense probe showing the earliest expression of *KCNQ*: that is, widespread throughout the nervous system, with little detectable expression elsewhere. **B.** No *KCNQ* expression was revealed in similarly aged *KCNQ* deletion mutant embryos stained with antisense probe. Wild-type embryos stained with the control sense probe (**C.**) and *KCNQ* deletion mutants hybridised with the sense probe (**D.**) showed little or no non-specific staining.
(PDF)

Figure S2 **Changes in DDC neuron KCNQ levels leads to a wing expansion defect associated with impaired release A.** Histogram showing that increasing or decreasing the level of KCNQ in Ddc neurons results in an increase (p<0.01) in the frequency of the wing expansion defect compared to control (*Ddc-Gal4, CSw-*). Data were analysed by 1-way ANOVA with a Bonferroni post-hoc test (n≥8, ∼20 flies per n).
(PDF)

Figure S3 **Ethanol does not cause a change in voltage-dependent activation of mammalian or** ***Drosophila*** **KCNQ channels.** The G–V relation for KCNQ2/KCNQ3 (**A.**) shows no apparent shift (p>0.05) by 10 mM ethanol (grey, *V_0.5_* = −10.6±1.6 mV; slope factor = 14.2±1.5 mV), compared to control (black, *V_0.5_* = −7.4±3.1 mV; slope factor = 17.2±3.1 mV). **B.** The dKCNQ control current (black, *V_0.5_* = −2.2±4.6 mV; slope factor = 20.5±2.9 mV) and the current in the presence of 10 mM ethanol (grey, *V_0.5_* = 11.5±1.0 mV; slope factor = 28.2±5.3 mV) show that the blocker does not cause a significant (p>0.05) shift in whole cell current activation. Activation relations were calculated from tail currents. Data were analysed by Student's paired *t*-test. n≥4. For all figures: error bars are standard error of the mean and no asterisk means not significant; *p<0.05, **p<0.01 and ***p<0.001.
(PDF)

Figure S4 **KCNQ signalling does not affect ethanol metabolism or avoidance.** **A.** The sedation assay was performed in the absence of ethanol in order to control for any possible non-specific locomotor effect caused by heating flies. In the absence of ethanol, flies do not become sedated, so the per cent sedated or T_50%_ sedated is not possible to calculate. Instead, the number of flies at the bottom of the bottle at a given time was counted. *Th-Gal4, +* (23°C, black square), *Th-Gal4, +* (30°C, white square), *Th-Gal4, uas-TRPA1* (23°C, black circle) and *Th-Gal4, uas-TRPA1* (30°C, white circle) were compared (n>9, 20 flies per n). **B.** Heat activation of TRPA1 in Th neurons did not predispose flies to sedation or cause a non-specific locomotor deficit. The number of *Th-Gal4, +* and *Th-Gal4, uas-TRPA1* flies at the bottom of the bottle was counted at 23 or 30°C (n>9, 20 flies per n) and 2-way ANOVA indicates that genotype and temperature did not affect this number (p>0.05). **C.** The ethanol content of experimental and control (*CSw-* wild-type and *KCNQ* control) genotypes were quantified using an alcohol-dehydrogenase-based assay, in which the absorption levels of all genotypes were similar (p>0.05) at the end of the 90 min exposure to 40% ethanol vapour. Likewise, the rate of catabolism as reflected by the ethanol content after 50 min recovery from the exposure was the same between genotypes. **D.** Experimental and control (*CSw-* wild-type, *KCNQ* control and *Elav-Gal4, +*) flies similarly (p>0.05) avoided the arm of the T-maze containing 40% ethanol. All data were analysed by 1-way ANOVA with Bonferroni post-hoc test.
(PDF)

## References

[1] Wulff H, Castle NA, Pardo LA (2009) Voltage-gated potassium channels as therapeutic targets. Nat Rev Drug Disc 8: 982–1001. 10.1038/nrd2983 PMC2790170 19949402

[2] Ashcroft FM (2006) From molecule to malady. Nature 440: 440–7. 16554803 10.1038/nature04707

[3] Littleton JT, Ganetzky B (2000) Ion channels and synaptic organization: analysis of the *Drosophila* genome. Neuron 26: 35–43. 10798390 10.1016/s0896-6273(00)81135-6

[4] Unoki H, Takahasi A, Kawaguchi T, Hara K, Horikoshi M, et al (2008) SNPs in KCNQ1 are associated with susceptibility to type 2 diabetes in East Asian and European populations. Nat Genet 40: 1098–102. 18711366 10.1038/ng.208

[5] Yasuda K, Miyake K, Horikawa Y, Hara H, Osawa H, et al (2008) Variants in *KCNQ1* are associated with susceptibility to type 2 diabetes mellitus. Nat Genet 40: 1092–1097. 18711367 10.1038/ng.207

[6] Koyama S, Brodie MS, Appel SB (2007) Ethanol inhibition of M-current and ethanol induced direct excitation of Ventral Tegmental Area dopamine neurons. J Neurophysiol 97: 1977–85. 16956995 10.1152/jn.00270.2006 PMC2372163

[7] Peters HC, Hu H, Pongs O, Storm JF, Isbrandt D (2005) Conditional transgenic suppression of M channel in mouse brain reveals functions in neuronal excitability, resonance and behaviour. Nat Neurosci 8: 51–60. 15608631 10.1038/nn1375

[8] Soldovieri MV, Miceli F, Taglialatatela M (2011) Driving with no brakes: molecular pathophysiology Kv7 potassium channels. Physiology 26: 365–76. 22013194 10.1152/physiol.00009.2011

[9] Ocorr K, Reeves NL, Wessells RJ, Fink M, Chen HS, et al (2007) *KCNQ* potassium channel mutations cause cardiac arrhythmias in *Drosophila* that mimic the effects of aging. Proc Natl Acad Sci USA 104: 3943–3948. 17360457 10.1073/pnas.0609278104 PMC1820688

[10] Wen H, Weiger TM, Ferguson TS, Shahidullah M, Scott SS, et al (2005) A *Drosophila* KCNQ channel essential for early embryonic development. J Neurosci 25: 10147–10156. 16267222 10.1523/JNEUROSCI.3086-05.2005 PMC6725806

[11] Cavaliere S, Hodge JJ (2011) *Drosophila* KCNQ channel displays evolutionarily conserved electrophysiology and pharmacology with mammalian KCNQ channels. PLoSONE 6: e23898. 10.1371/journal.pone.0023898 PMC3168433 21915266

[12] Atkinson NS (2009) Tolerance in *Drosophila* . J Neurogen 23: 293–302. 10.1080/01677060802572937 PMC4864953 19180359

[13] Kaun KR, Devineni AV, Heberlein U (2012) *Drosophila melanogaster* as a model to study drug addiction. Hum Genet 131: 959–75. 22350798 10.1007/s00439-012-1146-6 PMC3351628

[14] Hodge JJ, Mullasseril P, Griffith LC (2006) Activity-dependent gating of CaMKII autonomous activity by *Drosophila* CASK. Neuron 51: 327–337. 16880127 10.1016/j.neuron.2006.06.020

[15] Mahr A, Aberle H (2006) The expression pattern of the *Drosophila* vesicular glutamate transporter: a marker protein for motorneurons and glutamatergic centers in the brain. Gene Expr Patterns 6: 299–309. 16378756 10.1016/j.modgep.2005.07.006

[16] Tian L, Hires SA, Mao T, Huber D, Chiappe ME, et al (2009) Imaging neural activity in worms, flies and mice with improved GCaMP calcium indicators. Nat Methods 6: 875–881. 19898485 10.1038/nmeth.1398 PMC2858873

[17] Cheng LE, Song W, Looger LL, Jan LY, Jan YN (2010) The role of the TRP channel NompC in *Drosophila* larval and adult locomotion. Neuron 67: 373–380. 20696376 10.1016/j.neuron.2010.07.004 PMC2933178

[18] Wen T, Parrish CA, Xu D, Wu Q, Shen P (2005) *Drosophila* neuropeptide F and its receptor, NPFR1, define a signaling pathway that acutely modulates alcohol sensitivity. Proc Natl Acad Sci USA 102: 2141–2146. 15677721 10.1073/pnas.0406814102 PMC548536

[19] Pulver S, Pashkovski SL, Hornstein NJ, Garrity PA, Griffith LC (2009) Temporal dynamics of neuronal activation by channelrhodopsin-2 and TRPA1 determine behavioural output in *Drosophila* larvae. J Neurophysiol 101: 3075–88. 19339465 10.1152/jn.00071.2009 PMC2694103

[20] Hodge JJ, Choi JC, O'Kane CJ, Griffith LC (2005) *Shaw* potassium channel genes in *Drosophila* . J Neurobiol 63: 235–254. 15751025 10.1002/neu.20126

[21] Peabody NC, Pohl JB, Diao F, Vreede AP, Sandstrom DJ, et al (2009) Characterization of the decision network for wing expansion in *Drosophila* using targeted expression of the TRPM8 channel. J Neurosci 28: 14379–14391. 10.1523/JNEUROSCI.4241-08.2009 PMC2717795 19295141

[22] Hodge JJ (2009) Ion channels to inactivate neurons in *Drosophila* . Front Mol Neurosci 2: 13. 19750193 10.3389/neuro.02.013.2009 PMC2741205

[23] Nicola SM, Surmeier J, Malenka RC (2000) Dopaminergic modulation of neuronal excitability in the striatum and nucleus accumbens. Annu Rev Neurosci 23: 185–215. 10845063 10.1146/annurev.neuro.23.1.185

[24] Spanagel R (2009) Alcoholism: A systems approach from molecular physiology to addictive behaviour. Physiol Rev 89: 649–705. 19342616 10.1152/physrev.00013.2008

[25] Sulzer D (2011) How addictive drugs disrupt presynaptic dopamine neurotransmission. Neuron 69: 628–649. 21338876 10.1016/j.neuron.2011.02.010 PMC3065181

[26] Alekseyenko OV, Lee C, Kravitz EA (2010) Targeted manipulation of serotonergic neurotransmission affects the escalation of aggression in adult male *Drosophila melanogaster* . PLoS ONE 5: e10806. 20520823 10.1371/journal.pone.0010806 PMC2875409

[27] Kong EC, Woo K, Li H, Lebestky T, Mayer N, et al (2010) A pair of dopamine neurons target the D1-like dopamine receptor DopR in the central complex to promote ethanol stimulated locomotion in *Drosophila* . PLoS ONE 5: e9954. 20376353 10.1371/journal.pone.0009954 PMC2848596

[28] Kaun KR, Azanchi R, Maung Z, Hirsh J, Heberlein U (2011) A *Drosophila* model of alcohol reward. Nat Neuro 14: 612–621. 10.1038/nn.2805 PMC4249630 21499254

[29] Koob GF (2004) A role for GABA mechanisms in the motivational effects of alcohol. Biochem Pharmacol 68: 1515–25. 15451394 10.1016/j.bcp.2004.07.031

[30] Harris RA, Trudell JR, Mihic SJ (2008) Ethanol's molecular targets. Sci Signal 1: re7. 18632551 10.1126/scisignal.128re7 PMC2671803

[31] Covarrubias M, Rubin E (1993) Ethanol selectively blocks a non-inactivating K^+^ current expressed in *Xenopus* oocytes. Proc Natl Acad Sci USA 90: 19408–19416. 10.1073/pnas.90.15.6957 PMC47054 8346202

[32] Treistman SN, Martin GE (2009) BK channels: mediators and models for alcohol tolerance. TINS 32: 629–637. 19781792 10.1016/j.tins.2009.08.001 PMC4115799

[33] Morozova TV, Anholt RRH, Mackay TFC (2007) Phenotypic and transcriptional response to selection for alcohol sensitivity in *Drosophila melanogaster* . Genome Biology 8: R231. 17973985 10.1186/gb-2007-8-10-r231 PMC2246305

[34] Ogueta M, Cibik O, Eltrop R, Schneider A, Scholz H (2010) The influence of Adh function on ethanol preference and tolerance in adult *Drosophila melanogaster* . Chem Senses 35: 813–822. 20739429 10.1093/chemse/bjq084

[35] Bainton RJ, Tsai LT, Singh CM, Moore MS, Neckameyer WS, et al (2000) Dopamine modulates acute responses to cocaine, nicotine and ethanol in *Drosophila* . Curr Biol 10: 187–194. 10704411 10.1016/s0960-9822(00)00336-5

[36] Li H, Chaney S, Forte M, Hirsh J (2000) Ectopic G-protein expression in dopamine and serotonin blocks cocaine sensitization in *Drosophila melanogaster* . Curr Biol 10: 211–214. 10704417 10.1016/s0960-9822(00)00340-7

[37] Dzitoyeva S, Dimitrijevic N, Manev H (2003) γ-Aminobutyric acid B receptor 1 mediates behaviour-impairing actions of alcohol in *Drosophila*: adult RNA interference and pharmacological evidence. Proc Natl Acad Sci USA 100: 5485–90. 12692303 10.1073/pnas.0830111100 PMC154371

[38] Mezler M, Muller T, Raming K (2001) Cloning and functional expression of GABA_B_ receptors from *Drosophila* . Eur J Neurosci 13: 477–486. 11168554 10.1046/j.1460-9568.2001.01410.x

[39] Lewohl JM, Wilson WR, Mayfield RD, Brozowski SJ, Morrisett RA, et al (1999) G-protein-coupled inwardly rectifying K^+^ channels. Nat Neurosci 2: 1084–90. 10570485 10.1038/16012

[40] Hodge CW, Mehmert KK, Kelley SP, McMahon T, Haywood A, et al (1999) Supersensitivity to allosteric GABA(A) receptor modulators and alcohol in mice lacking PKCepsilon. Nat Neurosci 2: 997–1002. 10526339 10.1038/14795

[41] Mayfield RD, Harris RA, Schuckit MA (2008) Genetic factors influencing alcohol dependence. Br J Pharmacol 154: 275–87. 18362899 10.1038/bjp.2008.88 PMC2442454

[42] Hansen HH, Andreasen JT, Weikop P, Mirza N, Scheel-Krüger J, et al (2007) The neuronal KCNQ channel opener retigabine inhibits locomotor activity and reduces forebrain excitatory responses to the psychostimulants cocaine, methylphenidate and phencyclidine. Eur J Pharm 570: 77–88. 10.1016/j.ejphar.2007.05.029 17628530

[43] Kapfhamer D, Berger KH, Hopf FW, Seif T, Kharazia V, et al (2010) Protein phosphatase 2a and glycogen synthase kinase 3 signaling modulate prepulse inhibition of the acoustic startle response by altering cortical M-type potassium channel activity. J Neurosci 30: 8830–8840. 20592205 10.1523/JNEUROSCI.1292-10.2010 PMC3842471

[44] Hyman SE, Malenka RC, Nestler EJ (2006) Neural mechanisms of addiction: the role of reward-related learning and memory. Annu Rev Neurosci 29: 565–598. 16776597 10.1146/annurev.neuro.29.051605.113009

[45] Keene AC, Waddell S (2007) *Drosophila* olfactory memory: single genes to complex neural circuits. Nature Rev Neurosci 8: 341–354. 17453015 10.1038/nrn2098

[46] Lima SQ, Miesenböck G (2005) Remote control of behavior through genetically targeted photostimulation of neurons. Cell 121: 141–152. 15820685 10.1016/j.cell.2005.02.004

