# Supplementary figures and images for: KCNQ Channels Show Conserved Ethanol Block and Function in Ethanol Behaviour
Source: PLoS One. 2012 Nov 29;7(11):e50279. doi: 10.1371/journal.pone.0050279 (PMC3510227; doi:10.1371/journal.pone.0050279)

**Supporting Figure 1, Cavaliere et al.**

**A**

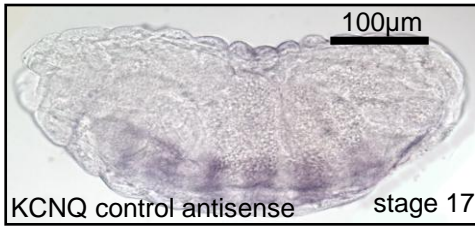

**B**

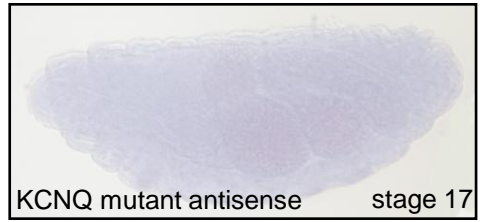

**C**

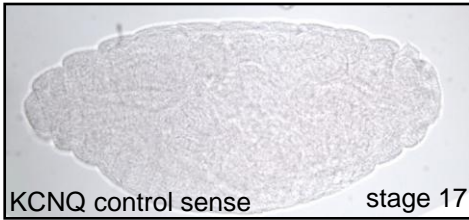

**D**

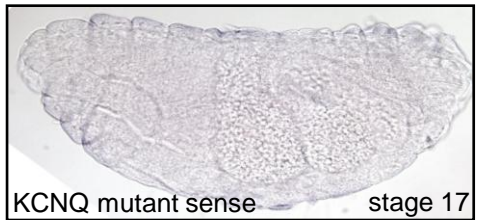

Supplement: Figure S1 — KCNQ is widely expressed in the nervous system. A. Stage 17 wild-type embryos hybridised with KCNQ antisense probe showing the earliest expression of KCNQ: that is, widespread throughout the nervous system, with little detectable expression elsewhere. B. No KCNQ expression was revealed in similarly aged KCNQ deletion mutant embryos stained with antisense probe. Wild-type embryos stained with the control sense probe (C.) and KCNQ deletion mutants hybridised with the sense probe (D.) showed little or no non-specific staining. (PDF) [file pone.0050279.s001.pdf]

Supporting Figure 2, Cavaliere et al.

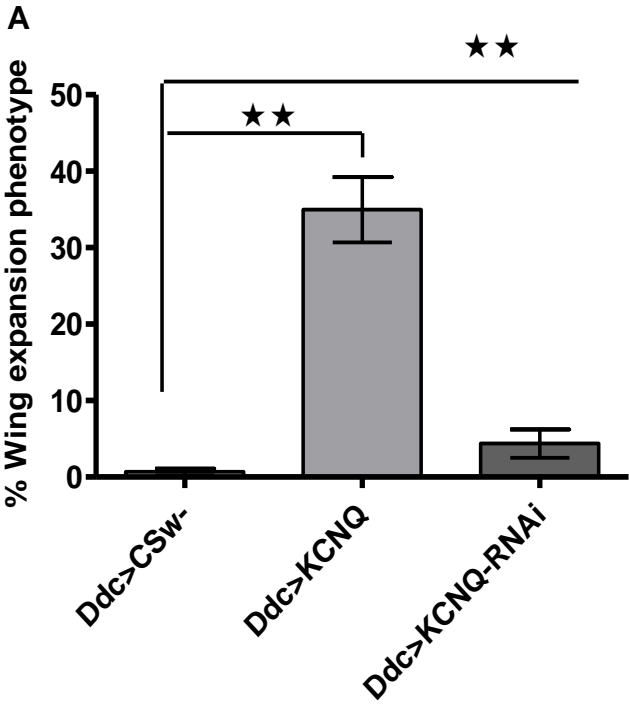

Supplement: Figure S2 — Changes in DDC neuron KCNQ levels leads to a wing expansion defect associated with impaired release A. Histogram showing that increasing or decreasing the level of KCNQ in Ddc neurons results in an increase (p<0.01) in the frequency of the wing expansion defect compared to control (Ddc-Gal4, CSw-). Data were analysed by 1-way ANOVA with a Bonferroni post-hoc test (n≥8, ∼20 flies per n). (PDF) [file pone.0050279.s002.pdf]

Supporting Figure 3, Cavaliere et al.

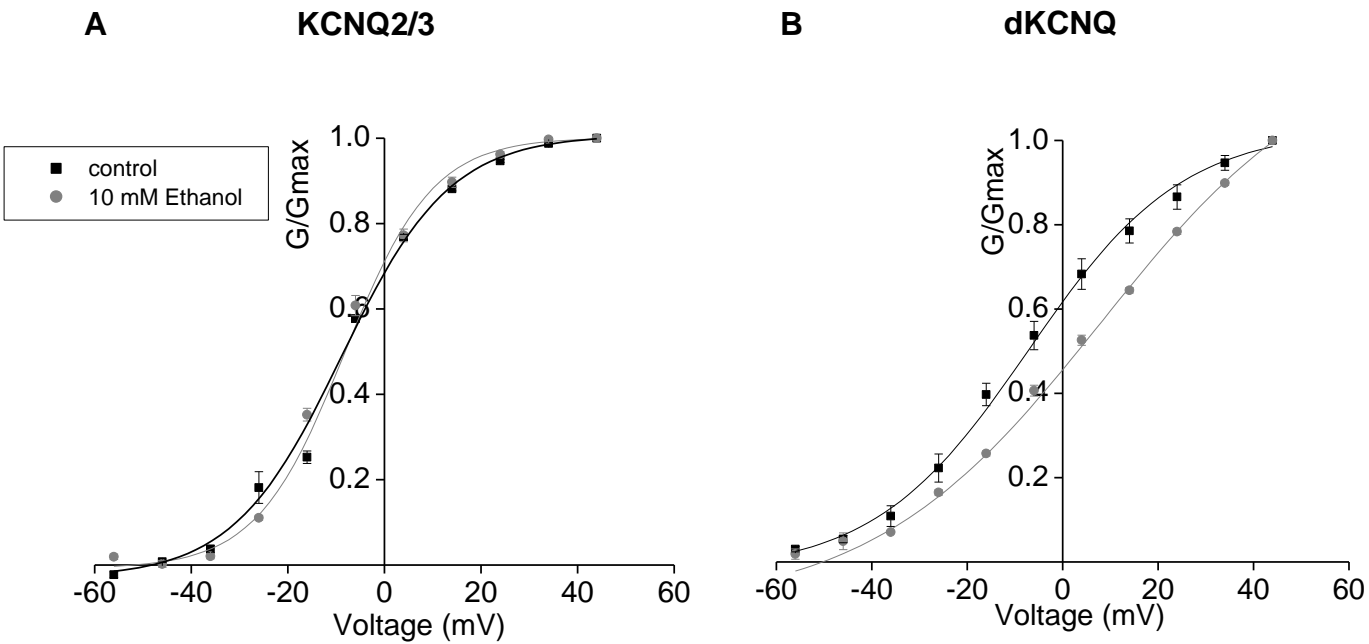

Supplement: Figure S3 — Ethanol does not cause a change in voltage-dependent activation of mammalian or Drosophila KCNQ channels. The G–V relation for KCNQ2/KCNQ3 (A.) shows no apparent shift (p>0.05) by 10 mM ethanol (grey, V0.5 = −10.6±1.6 mV; slope factor = 14.2±1.5 mV), compared to control (black, V0.5 = −7.4±3.1 mV; slope factor = 17.2±3.1 mV). B. The dKCNQ control current (black, V0.5 = −2.2±4.6 mV; slope factor = 20.5±2.9 mV) and the current in the presence of 10 mM ethanol (grey, V0.5 = 11.5±1.0 mV; slope factor = 28.2±5.3 mV) show that the blocker does not cause a significant (p>0.05) shift in whole cell current activation. Activation relations were calculated from tail currents. Data were analysed by Student's paired t-test. n≥4. For all figures: error bars are standard error of the mean and no asterisk means not significant; *p<0.05, **p<0.01 and ***p<0.001. (PDF) [file pone.0050279.s003.pdf]

Supporting Figure 4, Cavaliere et al.

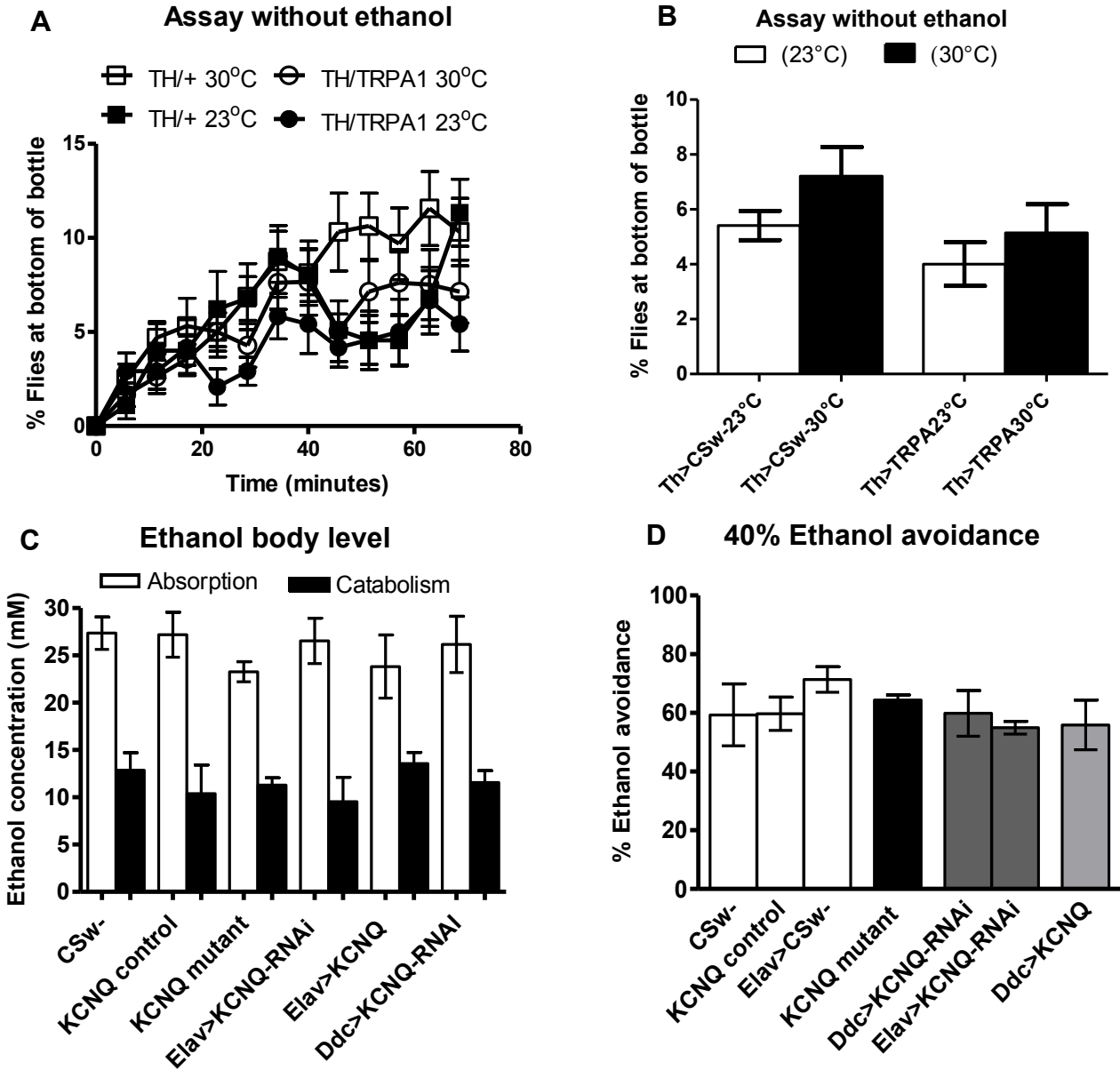

Supplement: Figure S4 — KCNQ signalling does not affect ethanol metabolism or avoidance. A. The sedation assay was performed in the absence of ethanol in order to control for any possible non-specific locomotor effect caused by heating flies. In the absence of ethanol, flies do not become sedated, so the per cent sedated or T50% sedated is not possible to calculate. Instead, the number of flies at the bottom of the bottle at a given time was counted. Th-Gal4, + (23°C, black square), Th-Gal4, + (30°C, white square), Th-Gal4, uas-TRPA1 (23°C, black circle) and Th-Gal4, uas-TRPA1 (30°C, white circle) were compared (n>9, 20 flies per n). B. Heat activation of TRPA1 in Th neurons did not predispose flies to sedation or cause a non-specific locomotor deficit. The number of Th-Gal4, + and Th-Gal4, uas-TRPA1 flies at the bottom of the bottle was counted at 23 or 30°C (n>9, 20 flies per n) and 2-way ANOVA indicates that genotype and temperature did not affect this number (p>0.05). C. The ethanol content of experimental and control (CSw- wild-type and KCNQ control) genotypes were quantified using an alcohol-dehydrogenase-based assay, in which the absorption levels of all genotypes were similar (p>0.05) at the end of the 90 min exposure to 40% ethanol vapour. Likewise, the rate of catabolism as reflected by the ethanol content after 50 min recovery from the exposure was the same between genotypes. D. Experimental and control (CSw- wild-type, KCNQ control and Elav-Gal4, +) flies similarly (p>0.05) avoided the arm of the T-maze containing 40% ethanol. All data were analysed by 1-way ANOVA with Bonferroni post-hoc test. (PDF) [file pone.0050279.s004.pdf]
